# Does Playing Experience Improve Coaching? An Exploratory Study of Perceptual-Cognitive Skill in Soccer Coaches

**DOI:** 10.3389/fpsyg.2013.00129

**Published:** 2013-03-21

**Authors:** Anna Gründel, Jörg Schorer, Bernd Strauss, Joseph Baker

**Affiliations:** ^1^Institute of Sport and Exercise Sciences, University of MuensterMünster, Germany; ^2^Kinesiology and Health Science, York UniversityToronto, ON, Canada

**Keywords:** coaching, expertise, pattern recognition, decision-making, sport

## Abstract

In many sports, it is common for top coaching positions to be held by former players; however, despite the natural progression in many sports for skilled players to become high level coaches, we have little understanding of how playing may develop useful skills for coaching. In this study we considered perceptual-cognitive skill across groups of high and low-skilled soccer players and soccer coaches. A range of perceptual-cognitive variables was measured in an attempt to capture the diverse skills related to expertise in sport and coaching. Generally, results highlighted similarities between coaches and players on some tasks and differences on others.

## Introduction

The path to coaching expertise is not well understood. On the one hand, there has been considerable research exploring the art and science of coaching, in particular the work of Smith and Smoll (see Smith and Smoll, 2007; Smoll and Smith, 2010 for reviews of this work). Most studies in this area consider the specific behaviors demonstrated by the expert coach (for example, see Horton et al., 2005), typically showing that expert coaching is characterized by meticulous planning of practices so that training reflects the specific needs and objectives of the athletes and team. On the other hand, although interesting, Abraham and Collins (1998) argue that this type of research has limited utility because it does not provide tangible information for improving coach development, particularly given the considerable time constraints involved in elite-level coaching where decisions often need to be made rapidly and accurately.

A limited number of studies have also considered the specific cognitive (as opposed to behavioral) qualities that define the expert coach. For instance, Vergeer and Lyle (2009) considered decision-making of gymnastic coaches to determine whether competency improved with increasing experience. They had coaches consider a specific scenario (i.e., participation of an injured athlete at a competition) and analyzed their responses. More skilled coaches showed more extensive and complex decision-making strategies, a higher number of statements and a deeper (i.e., less superficial) focus of attention when compared with less-skilled coaches. Studies such as these add to our understanding of the acquisition of coaching skill; however, our understanding is far from complete.

To complicate this discussion further, coaches have multiple roles in athlete training and development. Brack (2002) discussed the different requirements of team sport coaches noting three roles that a coach has to fill, each with its own specific competences. For example, in the “Trainer” role coaches have to demonstrate field competence while in the “Manager” role a coach needs to have strategic knowledge. Clearly, the role of the coach is multifaceted and variable, and understanding the process(es) of coach development is important for facilitating the development of the next generation of coaches.

In many sports, it is common for top coaching positions to be held by former players; however, despite this progression, we have little understanding of how *playing* may develop useful skills for *coaching*. Like other experts, skilled coaches appear to develop their capabilities over years of involvement. Schinke et al. (1995) suggested that in addition to developing through coaching experience, coach development could occur if they spent significant time as an athlete. Gilbert et al. (2006) examined the development profiles of successful coaches from a range of sports and competition levels, and presented preliminary data indicating these coaches had several 1000 h of accumulated time spent participating as an athlete (i.e., “pre-experience”). Subsequent to involvement as an athlete, they spent many 100 s of hours each year directly involved in coach-related activities (athlete training, competition, administration, and coach education; Gilbert et al., 2006). It is possible that participation as an athlete augments the development of skills necessary as an expert coach. Studies of athletes have provided a wealth of information about the skills underpinning elite performance in various sports (cf. Abernethy et al., 2007; Mann et al., 2007). Differences between skilled and less-skilled athletes have been found in components of *perception*, *knowledge*, and *decision-making* (Bar-Eli et al., 2011) and these skill differences may have some role in “creating” the expert coach. For example, the ability to rapidly read patterns of information on the field of play (a hallmark of skilled performance as an athlete; see Hodges et al., 2006, for a review) could be valuable for making quick decisions regarding the most effective offensive or defensive strategy. Unfortunately, few studies have considered the specific perceptual-cognitive capabilities of skilled coaches and how these relate to the capabilities demonstrated by skilled athletes. Understanding these relationships has the potential to improve our understanding of expert coach development and further elucidate the value of time spent as an athlete.

A range of methods has been used to uncover the difference in perceptual-cognitive capabilities of skilled athletes. For example, the Flicker-Paradigm, developed by Rensink et al. (1997), examines “change blindness,” a phenomenon where participants attempt to perceive subtle changes in a visual display. Experts typically perform better on these types of tasks than novices (cf. Werner and Thies, 2000) but only in scenes where information is structured in meaningful ways (cf. Werner and Thies, 2000; Reingold et al., 2001). Similar results have been found in a range of domains using various pattern recognition tests; differences between experts and novices are restricted to scenes with medium to high levels of structure (as compared to unstructured scenes; e.g., chess, Simon and Chase, 1973; snooker, Abernethy, 1994; soccer, Williams et al., 1993, 2006; Williams and Davids, 1998; Ward and Williams, 2003).

In addition to perceptual capabilities, skilled athletes have a more sophisticated *knowledge* *structure* than less-skilled athletes. For example, a series of studies by Allard and colleagues (see Allard et al., 1993 for a review) considered differences in declarative knowledge between coaches, players, and judges. They were able to show among athletes and judges/officials in figure skating, diving (e.g., Allard and Starkes, 1991), and basketball (e.g., Deakin and Allard, 1992) that different types of declarative knowledge are required for different sporting tasks. More specifically, declarative knowledge is specific to an individual’s role in the sport, differing between coaches, players, and judges.

An important distinction in understanding skilled performers’ *decision-making* processes relates to intuitive (i.e., automatic) versus deliberative (i.e., explicit information processing) decisions. Highly skilled athletes use both intuitive and deliberative processes; in high game tempo situations, they are able to make fast, intuitive, and accurate decisions, but when given the opportunity they are also able to deliberate this decision and propose other options (cf. Hogarth, 2001). Although there is some disagreement about the value of deliberation in decision-making (see Raab and Reimer, 2007), it appears that highly skilled athletes are superior at both intuitive and deliberative decision-making.

The research summarized above indicates skill-based differences among performers in dynamic, time-constrained sports. Given that elite coaches often spend a significant amount of time as athletes, it is important to determine whether the perceptual-cognitive skills developed by elite athletes over the course of their career have any relevance to being an exceptional coach. Our study considers differences on a range of perceptual-cognitive characteristics from varying levels of information processing among high- and low-skilled soccer coaches and high- and low-skilled players. Moreover, we considered whether the coaches’ skill in this area is explained by experience as a player. Because there are few studies on this topic, our hypotheses were rather preliminary. We assumed that there would be differences between coaches and players, particularly at higher levels of information processing (e.g., decision-making and strategizing; extended on the basis of Bar-Eli et al., 2011). Additionally, we hypothesized expertise differences at all levels of information processing. The interaction of both should become relevant in tasks that demand decisions and strategies but not for task of pure knowledge and perception.

## Materials and Methods

### Participants

A total of 73 males participated in this study, organized into two player and two coach groups. The “low-skilled players” group included 18 relatively inexperienced soccer players with an average of <300 h, SD = 388, and 1.9 years, SD = 2.4, of soccer playing experience and no coaching experience. The average age of participants in this group was 25.2 years, SD = 7.2. The “high-skilled players” group included 18 currently active soccer players of varying performance levels. They had played soccer in a club, typically at the amateur level, for an average of 3713 h, SD = 1648, and 17.2 years, SD = 3.0, demonstrating significant experience as a soccer player. Similar to the low-skilled player group, this group had no experience as a coach. The average age of participants in this group was 26.1 years, SD = 6.9.

The “low-skilled coaches” group consisted of 17 currently active soccer coaches with an average playing career of 5540 h, SD = 3558, and 23.5 years, SD = 7.4, with an average coaching career of 5160 h, SD = 3439, and 11.7 years, SD = 6.7. Participants in this group did not play at a level higher than the highest amateur level and were currently coaching at a low amateur level. The average age of this group was 40.0 years, SD = 9.3. The “high-skilled coaches” group was made up of 20 soccer coaches currently coaching at a high level. They had an average playing career of 5465 h, SD = 3408, and 23.0 years, SD = 8.9, generally at the highest amateur level, and a coaching career of 6670 h, SD = 4722, and 11.7 years, SD = 5.6, at the highest amateur level and above. Their average age was 35.5 years, SD = 9.8. All participants provided informed consent prior to the study and the study was conducted in accordance with the revised Ethical declaration of Helsinki (World Medical Association, 2008).

### Apparatus and procedure

A range of perceptual-cognitive variables was measured in an attempt to capture the diverse skills related to expertise in sport (Bar-Eli et al., 2011). All tests were presented on a notebook with a 15.1″ screen (IBM R31) and are described below.

#### Halftime speech test

Participants viewed a length of video from a 2006 game between a German first division team and one from the Turkish first division. This game was chosen because it was unlikely to be known by the participants. The play-by-play commentary was removed from the video. The participants’ task was to view the seven and a half minutes of play that occurred immediately before the halftime break. After viewing the segment, participants were instructed to put themselves in the position of head coach of the team that was zero to one behind at halftime, and deliver the halftime speech to their team. Speeches were recorded, transcribed, and evaluated using content analysis. A two-category system similar to that used by Hagemann et al. (2008) was used. Two language and sport experts assigned the statement of the participants to categories using the qualitative analysis technique of formal structuring (cf. Mayring, 2008). First, the addressee of the statement was considered to determine whether the statement was directed to an individual player, a group of players, or the whole team. Second, the content of the statement was considered to determine whether the content reflected instruction, anger/frustration, question, criticism, praise, motivation, or utterances without specific meanings. The frequency of utterances across these categories provided information about differences in the behavior among the four groups.

#### Forced-choice pattern recognition test

The ability to rapidly recognize offensive and defensive structures may be important for informing the type of complex and rapid decision-making necessary for high performance coaching. To this end, participants completed a forced-choice test immediately after the halftime speech. This test was used to determine participants’ skill at recognizing playing patterns. Participants viewed 10 scenes of 10 s, five of which were from the video they had just seen prior to the halftime speech task. The other five were from other parts of the same game. Any identifying information such as elapsed time, score, or commentary was deleted and the scenes were presented in random order. Participants were required to identify scenes they had seen previously with recognition performance measured as the number of correctly recognized scenes. To put the participants under time pressure, they only had 10 s time to make their decision.

#### Decision-making test

Our decision-making test utilized a similar approach to that described by Johnson and Raab (2003) for handball and can be classified in the family of Guilford tests (Christensen et al., 1960; Berger and Guilford, 1969). The test consisted of 15 (12 test plus 3 training) medium and high structured video scenes taken from four games from the German first division in 2006. Scenes were filmed from the perspective of the middle line high up in the ranks and the number of players in the scene ranged from 10 to 18. Three videos were used for familiarization with the task and the others were presented in random order to the coaches. Participants were provided with a portable recorder and a microphone and their answers were immediately transcribed. After viewing approximately 10 s of video the screen “froze” at a point where a decision had to be made. At this point, participants provided one quick first decision (=intuition) followed by generation of as many realistic options as possible (=option generation). After the last option was named, they were asked to identify which one they would see as the best option (=deliberation). Participants’ decisions were rated on a scale from 1 to 10 by two independent expert soccer coaches. One had been working as a coach for 17 years and had the second highest coaching license in Germany, while the other had 7 years of experience and the third highest coaching license. The average of these rankings determined the quality of the options, which allowed for the examination of not only the number of total options and the quality of first and best options but also the consistency between first and best options.

#### Pattern-recall test

The pattern-recall task consisted of 18 scenes with durations between 8 and 12 s, with the number of players displayed ranging from 7 to 13. Participants were instructed that they would see short videos and that their task was to remember the location of all players from the very last frame presented. Following the video segment, participants saw a blank white screen and the task was to designate the last locations of players from the team using the left button of the mouse for one team and the right button for the other team. They were told to be as exact as possible on the location of the players as they were last seen in the video. If they thought they made a mistake, they were able to easily erase and relocate this player on the screen. After they were done with one video, they started the next. No time pressure was included and accuracy was determined as the number of players recalled compared to the number of players presented on screen.

#### Flicker-test

The Flicker-test is a common method of exploring change blindness capabilities within a scene (cf. Rensink et al., 1997). Eighteen scenes from first division German soccer games were manipulated after approximately 10 s of video. At this point, the video frame froze then changed to a black screen before returning to a frame that appeared identical to the previous; the only difference between first frame and the next was that one player was removed using spatial occlusion. The task for the participants was to point out the player removed from the scene.

### Procedure

Data collection took between 1.5 and 2 h per participant. To get participants used to the Flicker and pattern-recall tests familiarization trails were used before each of these tests. The test elements were counterbalanced to avoid serial order effects and data collection took place in various quiet locations where participants could complete the tasks without interruption.

### Statistical analyses

Statistical analyses were conducted separately for each dependent variable. Analyses of variance were conducted to test for differences between groups with the more liberal Tukey-procedure used for *post hoc* analyses. For two tests (Flicker and Pattern-recall), structure was added as the repeated measure. Greenhouse–Geisser-adjustments were used when the precondition of variance homogeneity was violated and the alpha-level was set to 0.05 for all analyses. Both alpha-level and effect size (*f*) were used when interpreting differences among the groups. SPSS 18.0 and G*Power 3.1.10 (Faul et al., 2007) were used for all analyses.

## Results

The results are generally presented in the order of the type of information processing that occurred during the task. We start with the Flicker-test, followed by the forced-choice-tests, pattern-recall test, and decision-making test, ending with results from the halftime speech.

### Flicker-test

A between groups (groups) repeated measures (structure) ANOVA revealed significant differences for structure, *F*(2, 69) = 75.22, *p* < 0.01, *f* = 1.04, and group *F*(3, 69) = 3.46, *p* = 0.02, *f* = 0.39. As seen in Table 1, reaction times increased with less structure. *Post hoc* tests demonstrated significant differences between the low structured scenes and the medium, *D* = 3345.4, *p* = 0.02, and high structured ones, *D* = 3067.9, *p* = 0.02, while the medium and highly structured scenes did not differ. For the group main effect, *post hoc* tests showed differences only between the high-skilled players and the low-skilled coaches, *D* = 1656.4, *p* = 0.02. No significant interaction between the factors was revealed, *F*(6, 138) = 1.48, *p* = 0.19, *f* = 0.25, 1−β = 0.29.

**Table 1:** **Differences among player and coach groups on the Flicker-test**.

Characteristic	Low-skilled player		High-skilled player		Low-skilled coaches		High-skilled coaches	
	*M*	SD	*M*	SD	*M*	SD	*M*	SD
High structured scenes	4961	342	4766	342	5473	352	4983	325
Medium structured scenes	5168	372	4605	372	6111	383	5459	353
Low structured scenes	7802	707	7305	707	10061	727	7779	670

### Forced-choice pattern recognition test

**Figure 1:**
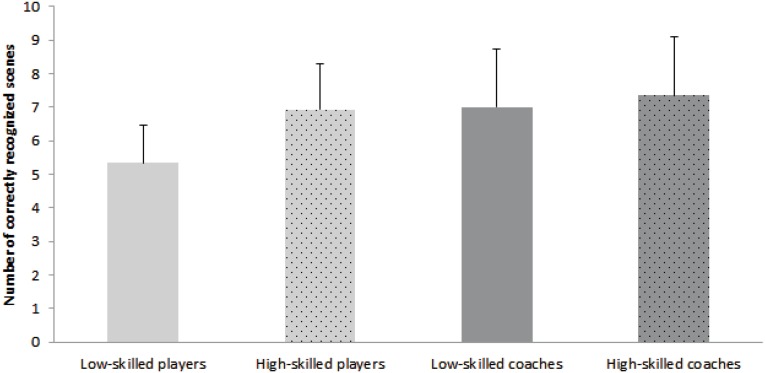
**Differences between player and coach groups on number of correctly recognized scenes (and their standard error) in the Forced-Choice-test**.

For the forced-choice pattern recognition test, significant differences between groups were revealed, *F*(3, 72) = 6.43, *p* < 0.01, *f* = 0.46. As seen in Figure 1, there were clear differences between the less-skilled players and the other three groups. *Post hoc* tests confirmed differences between less-skilled players and high-skilled players, *D* = 1.61, *p* = 0.01, less-skilled players and less-skilled coaches, *D* = 1.67, *p* = 0.01, and less-skilled players and high-skilled coaches, *D* = 2.02, *p* < 0.01, but did not show any difference between the three other groups.

### Pattern-recall test

**Figure 2:**
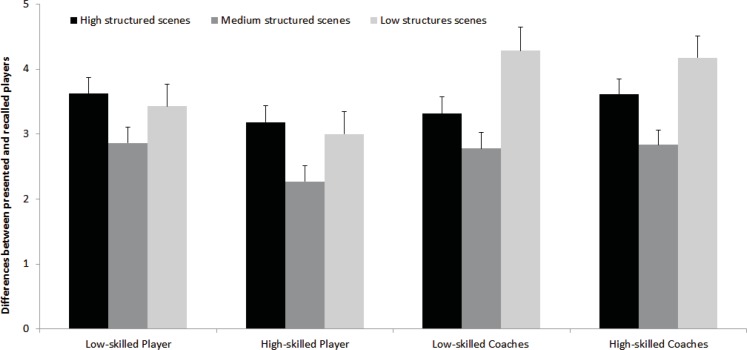
**Differences between player and coach groups in scenes with varying structure on number of players reported (and their standard error) in the Pattern-Recall test**.

A between subject (group) repeated measures (structure) ANOVA revealed a significant interaction between both factors, *F*(5.57, 128.19) = 3.31, *p* < 0.01, *f* = 0.39, as well as a main effect for structure, *F*(1.86, 128.19) = 40.99, *p* < 0.01, *f* = 0.77, but showed no significant differences between groups, *F*(3, 69) = 1.72, *p* = 0.17, *f* = 0.27, 1−β = 0.45 on the pattern-recall test. Repeated within-subject contrasts of the interaction between group and structured revealed the interaction was between medium and low structure scenes, *F*(3, 69) = 3.39, *p* = 0.02, but not between medium and high structured scenes, *F*(3, 69) = 0.57, *p* = 0.63. As can be seen in Figure 2, the coaches showed deteriorated performance in low structure scenes compared to the players.

### Decision-making test

In the decision-making test (Table 2), significant differences between groups were revealed in the number of options generated, *F*(3, 72) = 10.63, *p* < 0.01, *f* = 0.57. The difference between both of the player groups and both of the coaches groups was significant. The low-skilled players differed from the low-skilled coaches, *D* = 0.46, *p* < 0.01 and from the high-skilled coaches, *D* = 0.31, *p* < 0.01. Similarly, the high-skilled players differed from the low-skilled coaches, *D* = 0.41, *p* < 0.01, as well as from the high-skilled coaches *D* = 0.27, *p* = 0.02. Focusing on the quality of the options, there was neither a significant difference in the quality of the first choice between the groups, *F*(3, 69) = 0.74, *p* = 0.53, *f* = 0.18, 1−β = 0.21, nor in the quality of the final choice between the groups, *F*(3, 69) = 2.25, *p* = 0.09, *f* = 0.31, 1−β = 0.57, although in this latter case, the effect size was of moderate strength and the alpha value was approaching significance. A significant difference in the mean quality of all options was detected, *F*(3, 72) = 3.26, *p* = 0.03, *f* = 0.35, but the *post hoc* tests showed no significant differences between the groups. The consistency between the First and Final choice was also not significant, *F*(3, 69) = 0.38, *p* = 0.77, *f* = 0.13, 1−β = 0.12.

**Table 2:** **Differences among player and coach groups on the decision-making task**.

Characteristic	Low-skilled player		High-skilled player		Low-skilled coaches		High-skilled coaches	
	*M*	SD	*M*	SD	*M*	SD	*M*	SD
Number of options	2.0	0.3	2.1	0.3	2.5	0.4	2.3	0.4
Quality of the first choice	7.2	1.0	7.6	0.6	7.4	0.9	7.5	0.7
Quality of the final choice	7.0	1.0	7.5	0.7	7.9	1.0	7.6	0.6
Average option quality	6.2	0.6	6.7	0.4	6.6	0.4	6.6	0.4
Consistency between first choice and final choice	0.8	0.2	0.8	0.1	0.8	0.2	0.7	0.2

*The quality of the generated options was ranked by two experts on a scale from 1 – not recommended to 10 – optimal option*.

### Halftime speech test

The results of the analysis of the halftime speeches showed significant differences in both analyzing steps (Table 3). The word counts between the expertise groups differed significantly, *F*(3, 72) = 6.79, *p* < 0.01, *f* = 0.47, and *post hoc* tests showed that the low-skilled players differed from both groups of coaches. The difference between the low-skilled players and the low-skilled coaches was *D* = 170.3, *p* = 0.01, and the difference between the low-skilled players and the high-skilled coaches was *D* = 188.9, *p* < 0.01. Also, the number of statements was significantly different between the four expertise groups, *F*(3, 73) = 4.00, *p* = 0.01, *f* = 0.38. Here the low-skilled players had a significantly lower number of statements than the high-skilled players, *D* = 4.56, *p* = 0.04, the low-skilled coaches, *D* = 5.12, *p* = 0.02, and the high-skilled coaches, *D* = 4.55, *p* = 0.04.

**Table 3:** **Differences among player and coach groups on the halftime speech task**.

Characteristic	Low-skilled player		High-skilled player		Low-skilled coaches		High-skilled coaches	
	*M*	SD	*M*	SD	*M*	SD	*M*	SD
Word count	150.7	70.6	216.8	102.5	321.0	210.8	339.6	164.1
Statement count	8.0	4.1	12.6	4.9	13.1	5.8	12.6	5.3

Instruction	2.3	0.6	3.7	0.6	3.8	0.6	3.3	0.5
Anger	0.2	0.1	0.3	0.1	0.2	0.1	0.1	0.1
Question	0.0	0.1	0.3	0.1	0.2	0.1	0.3	0.1
Information	1.1	0.2	0.8	0.2	1.0	0.3	1.1	0.2
Criticism	1.7	0.5	3.2	0.5	5.5	0.5	5.6	0.5
Praise	0.7	0.3	0.9	0.3	0.6	0.3	0.5	0.2
Motivation	0.6	0.2	1.7	0.2	0.5	0.2	0.3	0.2
Talking	1.4	0.3	1.6	0.3	1.4	0.3	1.5	0.3

Individual player	0.4	0.3	1.2	0.3	1.2	0.3	1.1	0.3
Group of players	1.5	0.5	1.6	0.5	2.1	0.5	2.7	0.4
Team	6.1	1.0	9.7	1.0	9.8	1.0	8.8	0.9

*The first section of the table describes the number of words and statements. The second section provides information about the average number of statements from each type and the third section, the average number of statements directed to each of the different categories of addressee*.

The analyses of the addressors of the statements show significant differences in the Greenhouse–Geisser test, *F*(2, 69) = 182.30, *p* < 0.01, *f* = 1.08. Most comments were addressed to the team, followed by groups of players and then individual players. There was an interaction between the participant groups and the addressors, *F*(2, 138) = 182.27, *p* < 0.01, *f* = 0.48. Every group addressed the team most often but the low-skilled coaches and the high-skilled players had a greater number of statements addressed to the team than the other two groups. For the statements addressed to groups of players, high-skilled coaches addressed them most often followed by the low-skilled coaches and the high-skilled players, with low-skilled players addressing groups of players the least. Interestingly, low-skilled coaches did not address individual players very often. High-skilled players and low- and high-skilled coaches had about the same number of statements addressed to an individual player.

There were similar results for the types of utterances. There was a significant difference between the eight types of utterances, *F*(7, 69) = 75.2, *p* < 0.01, *f* = 1.08, and an interaction between the type of utterance and the participant groups, *F*(10.66, 245.12) = 5.39, *p* < 0.01, *f* = 0.48. Perhaps the most interesting result was the number of statements belonging to the criticism category. The coaches, in particular the high-skilled coaches, criticized more than the other groups, whereas praise was used more often by the two player groups. Motivational statements were used more by the high-skilled players than all other groups. Another interesting result related to the instruction category, which was used more often by low-skilled coaches and high-skilled players than by high-skilled coaches and low-skilled players. Results for the other statement categories were similar across the four groups.

## Discussion

This study examined the perceptual-cognitive abilities of soccer coaches and players of different skill levels. Our exploratory hypotheses were that there would be differences between players and coaches as well as between expertise levels across the different information processing outcomes. Generally, results highlighted similarities between coaches and players on some tasks and differences (particularly compared to low-skilled players) on others. For example, there were no significant differences between the coach and player groups on pattern-recall or on the mean quality of decisions made during the decision-making task. Conversely, in the pattern recognition test, low-skill players differed from high-skill players and both groups of coaches. As in previous research (cf. Ward and Williams, 2003), there was some evidence that the number of correctly recognized scenes increased with increasing skill but, surprisingly, high-skill players were similar to the coaching groups, which did not differ.

Collectively, these results indicate some similarities between the perceptual and cognitive skills used by athletes and those used by coaches. On the one hand, the similarities between both groups of coaches and the high-skill players suggests there may be some transfer between skills developed as a player and those required for coaching, although this finding requires further exploration. It is possible that these perceptual-cognitive skills are “carryovers” from their athlete careers; Schorer and Baker (2009) found remarkable stability of perceptual skills in athletes despite years of non-involvement. All of our coaches had considerable pre-experience playing soccer, and as a result it was not possible with the current design to determine whether these skills carryover from their playing career or whether they result from coaching experience. To overcome this issue, future studies should try to utilize coaches with no prior playing experience in the respective domain. This was not possible in the current study, perhaps due to the unique nature of development of German soccer coaches.

On the Flicker-test, low-skilled coaches were significantly different from high-skilled players, but differences in accuracy and reaction time were not as large as expected. It is possible that our test was too easy (all groups had very high accuracy), therefore not providing sufficient challenge to the groups so that more skilled performers could demonstrate superiority. In the reaction time data, there was better performance in segments with increased structure, supporting previous research (Hodges et al., 2006) indicating a role for structure in information recall. However, differences among the groups were not affected by changes in structure, which is counter to our hypothesis that more skilled performers would be markedly superior to less-skilled performers in scenes with more structure. The high-skilled players had the shortest reaction time, but low-skilled players were similar to high-skilled coaches, with low-skilled coaches as the slowest group. These results are contrary to those reported be Werner and Thies (2000), which showed lower change blindness in experts. It is possible that the variation between the present results and those of Werner and Thies are due to the different ages of the groups in our study. The group with the slowest reaction time was also the oldest (40.0 years) and given the strong correlation between reaction time and age (in the present study *r* = −0.82) age may have confounded our analyses. If possible, future studies should consider re-examining these relationships with groups that are homogenous for age. Given the natural progression in many sports where coaching careers happen after careers of varying lengths as a player, this recommendation, although valuable for improving study design, is likely impossible to achieve.

There were also some clear differences between player and coach groups. In the decision-making task, coaches reported more options than players but there were no differences between the skill groups. Similarly, low-skill players had a lower word count than both coach groups on the halftime speech task. There was also a difference between low-skill players and low-skill coaches on the number of statements on this task. Additionally, coaches’ better performances on aspects of the halftime speech highlight the specific domains of expertise distinguishing coaching skill. Surprisingly, there were few differences between the high- and low-skill coaching groups, but when they did occur, they were typically on variables more closely associated with coaching skill such as the halftime task.

Taken together, these preliminary results allow us to develop a working hypothesis for future work. It seems that in lower levels of information processing, such as the more perceptual tasks, players outperform coaches, especially when the coaches have little or less qualified experience as players, while tasks with higher demands on information processing, like strategizing or decision-making, are more specifically related to coaching skill. Although this is obviously quite preliminary, it seems plausible that players would use more implicit ways of information processing in contrast to coaches who need to be very explicit in their processing. Future studies should test these constructs in addition to the already proposed comparison groups.

Although this study provides important insight into the qualities of skilled coaches and how these factors are different or similar to those of skilled athletes, several methodological issues remain. First, how do we determine the relevance of different forms of experience to the development of expert coaches? The average years of coaching experience of highly skilled coaches in the current study was 11.7, SD = 5.6 with an average of 6670 h of coaching experience; however, if we include time as a player, years of experience increases to 35 and the hours of experience (i.e., playing and coaching) increases to 12135 h, SD = 6002.6. Most models of skill acquisition focus on the role of domain-specific forms of training/practice (e.g., deliberate practice); the results of this study suggest that other forms of training are relevant for acquisition of coaching skill. Moreover, within the field of coaching there are various roles individuals can fill (e.g., assistant coach, technical coach), with head coach likely representing an elite stage of coach development. Currently, we have very little understanding of the value associated with time spent in junior coaching positions. A second, but related issue is the difficulty in determining the “quality” of a coach. The indicators mentioned above provide very gross indicators of skill development but coach quality or skill is often determined by other factors. For instance, the performances of athletes under a coach’s supervision are presumed to reflect a coach’s capability; similarly, a coach’s level of certification (e.g., the level of their coaching license in the present study) gives another indicator of coach “quality.” Unlike high performance athletes where there are clear performance standards for determining level of skill or quality of performance, there is no standard definition of coaching expertise, and as a result it is difficult to determine whether the differences in experience, performance level and qualification between the low- and high-skilled coaches are sufficient to call the high-skilled coaches “experts.”

A final methodological issue relates to the external validity of the study results. For example, the videos used during data collection were from television footage, where the camera position was on the side of the court or close to the center-line. This perspective is not normally used by either coach or athlete, which may limit the ecological validity of the study’s results. The negative effect of a changing viewing position was previously noted in a decision-making-soccer-test (Petit and Ripoll, 2008) and as a result, further research is necessary to confirm these findings in other samples using different methodologies.

Collectively, these results highlight coach- and player-specific perceptual-cognitive capabilities. Moreover, they also showed that recognition performance in the Forced-Choice-test and aspects of the halftime speech were specifically related to coaching skill. These results provide a useful starting point for further research examining aspects of perceptual-cognitive expertise in coaching.

## Conflict of Interest Statement

The authors declare that the research was conducted in the absence of any commercial or financial relationships that could be construed as a potential conflict of interest.

## References

[B1] Abernethy B. (1994). “The nature of expertise in sport,” in International Perspectives on Sport and Exercise Psychology, eds Sepra S. Alves J. Pataco V. (Morgantown: Fitness Information Technology), 57–68

[B2] Abernethy B. Maxwell J. P. Jackson R. C. Masters R. S. W. (2007). “Skill in sport,” in Handbook of Applied Cognition, 2nd Edn, eds Durso F. Nickerson R. Dumais S. Lewandowsky S. Perfect T. (New York: Wiley), 333–359

[B3] Abraham A. Collins D. (1998). Examining and extending research in coach development. Quest 50, 59–79 10.1080/00336297.1998.10484264

[B4] Allard F. Deakin J. M. Rogers W. M. (1993). “Declarative knowledge in skilled motor performance: byproduct or constituent?” in Cognitive Issues in Motor Expertise, eds Starkes J. L. Allard F. (Amsterdam: Elsevier), 95–108

[B5] Allard F. Starkes J. L. (1991). “Motor-skill experts in sports, dance, and other domains,” in Toward a General Theory of Expertise, eds Ericsson K. A. Smith J. (Cambridge: Cambridge University Press), 126–152

[B6] Bar-Eli M. Plessner H. Raab M. (2011). Judgment, Decision-Making and Success in Sport. Hoboken, NJ: Wiley-Blackwell

[B7] Berger R. M. Guilford J. P. (1969). Plot Titles. Beverly Hills: Sheridan Psychological Services

[B8] Brack R. (2002). Sportspielspezifische Trainingslehre. Wissenschafts- und objekttheoretische Grundlagen am Beispiel Handball. Hamburg: Czwalina

[B9] Christensen P. A. Guilford J. P. Merrifield P. R. Wilson R. C. (1960). Alternate Uses. Beverly Hills: Sheridan Psychological Services

[B10] Deakin J. M. Allard F. (1992). An evaluation of skill and judgement in basketball officiating. Paper Presented at the Meeting of the North American Society for the Psychology of Sport and Physical Activity, Pittsburgh, PA

[B11] Faul F. Erdfelder E. Lang A.-G. Buchner A. (2007). G*power 3: a flexible statistical power analysis program for the social, behavioral, and biomedical sciences. Behav. Res. Methods 39, 175–191 10.3758/BF03193146 17695343

[B12] Gilbert W. Côté J. Mallett C. (2006). Developmental paths and activities of successful sport coaches. Int. J. Sports Sci. Coach. 1, 69–76 10.1260/174795406776338526

[B13] Hagemann N. Strauss B. Büsch D. (2008). The complex problem-solving competence of team coaches. Psychol. Sport. Exerc. 9, 301–317 10.1016/j.psychsport.2007.04.003

[B14] Hodges N. J. Starkes J. L. MacMahon C. (2006). “Expert performance in sport: a cognitive perspective (Chapter 27),” in The Cambridge Handbook of Expertise and Expert Performance, eds Ericsson K. A. Charness N. Feltovich P. J. Hoffman R. R. (Cambridge: Cambridge University Press), 471–488

[B15] Hogarth R. M. (2001). Educating Intuition. Chicago, IL: University of Chicago Press

[B16] Horton S. Baker J. Deakin J. (2005). Experts in action: a systematic observation of 5 national team coaches. Int. J. Sport Psychol. 36, 299–319

[B17] Johnson J. Raab M. (2003). Take the first: option generation and resulting choices. Organ. Behav. Hum. Decis. Process 91, 215–229 10.1016/S0749-5978(03)00027-X

[B18] Mann D. T. Y. Williams A. M. Ward P. Janelle C. M. (2007). Perceptual-cognitive exercise in sport: a meta-analysis. J. Sport Exerc. Psychol. 29, 457–478 17968048 10.1123/jsep.29.4.457

[B19] Mayring P. (2008). Qualitative Inhaltsanalyse, Grundlagen und Techniken (10. Auflage). Weinheim: Beltz

[B20] Petit J. P. Ripoll H. (2008). Scene perceptions and decision making in sport simulation: a masked priming investigation. Int. J. Sports Psychol. 39, 1–19

[B21] Raab M. Reimer T. (2007). “Intuitive und deliberative Entscheidungen als Grundlage sportlicher Expertise,” in Psychologie der Sportlichen Höchstleistung: Grundlagen und Anwendung der Expertiseforschung, eds Hagemann N. Tietjens M. Strauss B. (Göttingen: Hogrefe), 93–117

[B22] Reingold E. M. Charness N. Pomplun M. Stampe D. M. (2001). Visual span in expert chess players: evidence from eye movements. Psychol. Sci. 12, 49–56 10.1111/1467-9280.00309 11294228

[B23] Rensink R. A. O’Regan J. K. Clark J. J. (1997). To see or not to see: the need for attention to perceive changes in scenes. Psychol. Sci. 8, 368–373 10.1111/j.1467-9280.1997.tb00427.x

[B24] Schinke R. J. Bloom G. A. Salmela J. H. (1995). The evolution of elite Canadian basketball coaches. Avante 1, 48–62

[B25] Schorer J. Baker J. (2009). An exploratory study of aging and perceptual-motor expertise in handball goalkeepers. Exp. Aging Res. 35, 1–19 10.1080/03610730802544641 19173099

[B26] Simon H. A. Chase W. G. (1973). Skill in chess. Am. Sci. 61, 394–403

[B27] Smith R. E. Smoll F. L. (2007). “Social-cognitive approach to coaching behaviors,” in Social Psychology in Sport, eds Jowett S. Lavallee D. (Champaign, IL: Human Kinetics), 75–90

[B28] Smoll F. L. Smith R. E. (2010). “Conducting psychologically oriented coach-training programs: a social-cognitive approach,” in Applied sport Psychology: Personal Growth to Peak Performance, 6th Edn, ed. Williams J. M. (Boston: McGraw-Hill), 392–416

[B29] Vergeer I. Lyle J. (2009). Coaching experience: examining the role in coaches decision making. Int. J. Sport Exerc. Psychol. 7, 431–449 10.1080/1612197X.2009.9671918

[B30] Ward P. Williams A. M. (2003). Perceptual and cognitive skill development in soccer: the multidimensional nature of expert performance. J. Sport Exerc. Psychol. 25, 93–111

[B31] Werner S. Thies B. (2000). Is “change blindness” attenuated by domain-specific expertise? An expert-novices comparison of change detection in football images. Vis. Cogn. 7, 163–173 10.1080/135062800394748

[B32] Williams A. M. Davids K. (1998). Visual search strategy, selective attention, and expertise in soccer. Res. Q. Exerc. Sport 69, 111–128 10.1080/02701367.1998.10607661 9635326

[B33] Williams A. M. Davids K. Burwitz L. Williams J. G. (1993). Cognitive knowledge and soccer performance. Percept. Mot. Skills 76, 579–593 10.2466/pms.1993.76.2.579

[B34] Williams A. M. Hodges N. North J. Barton G. (2006). Perceiving patterns of play in dynamic sport tasks: investigating the essential information underlying skilled performance. Perception 35, 317–332 10.1068/p5310 16619949

[B35] World Medical Association. (2008). Declaration of Helsinki – Ethical Principles for Medical Research Involving Human Subjects. Available at: http://www.wma.net/en/30publications/10policies/b3/index.html [accessed February 14, 2012].

